# Psychotherapy Practice Among Child and Adolescent Psychiatrists in the Philippines

**DOI:** 10.1192/j.eurpsy.2026.11735

**Published:** 2026-07-31

**Authors:** M. D. Chin

**Affiliations:** Section of Child and Adolescent Psychiatry, The Medical City, Pasig City, Philippines

## Abstract

**Introduction:**

Psychotherapy is a core skill in child and adolescent psychiatry practice. Psychotherapy remains an essential treatment modality for a child’s cognitive, emotional, behavioral, and relational problems. The knowledge and skills from psychotherapy contribute to other clinical activities such as diagnostic assessment, pharmacotherapy, and consultation to agencies, schools, and other physicians.

**Objectives:**

This study aimed to determine the patterns of psychotherapy among child and adolescent psychiatrists in the Philippines.

**Methods:**

Using a cross-sectional descriptive study design, a self-reported questionnaire was sent via email to each participant along with the informed consent. Out of 56 actively practicing child and adolescent psychiatrists in the Philippines, 49 child and adolescent psychiatrists fulfilled the inclusion criteria and were included in the study from November 2024 until July 2025. Data on demographic profile, current workload and practice, psychosocial interventions, conceptual frameworks, psychotherapy practices, and obstacles faced in doing psychotherapy were analyzed using descriptive statistics.

**Results:**

Based on the results, majority of the child and adolescent psychiatrists actively practicing in the Philippines were female (89.8%), aged 31–40 years old (34.7%) and living in Luzon area (73.5%). Most of the respondents have been practicing for less than 10 years (44.9%) in both private and government settings (59.2%) with increased outpatient load. Commonly, psychotropic medications (59.2%) with psychosocial interventions specifically in counseling (57.1%), parenting advice and guidance (75.5%), and psychotherapy (53.1%) are being practiced. In doing case formulation and treatment planning, attachment theory (51.1%) and learning and cognitive theory (42.9%) were frequently used. Supportive psychotherapy (77.6%) and cognitive behavioral therapy (46.9%) were most utilized in their practice, particularly for patients with depressive (100%) and anxiety disorders (98%). The main hindrance in providing psychotherapy is attributed to the number of duties and responsibilities practitioners handle (59.2%).

**Conclusions:**

In conclusion, it is of great importance that mental health workforce must be strengthened through (a) the increase of recruitment of trainees in child and adolescent psychiatry, (b) strengthen the capacity of general psychiatrists and non-psychiatrists such pediatricians/family practitioners to treat child and adolescent mental health problems, and (c) reinforce relationships with allied mental health professionals that can provide counseling and psychotherapy interventions such as guidance counselors and psychologists.

**Disclosure of Interest:**

None Declared

